# Uncertainty quantification of bioassay functions for the internal dosimetry of radioiodine

**DOI:** 10.1093/jrr/rraa081

**Published:** 2020-09-15

**Authors:** Tae-Eun Kwon, Yoonsun Chung, Jaeryong Yoo, Wi-Ho Ha, Minsu Cho

**Affiliations:** National Radiation Emergency Medical Center, Korea Institute of Radiological and Medical Sciences, 01812, Seoul, Republic of Korea; Department of Nuclear Engineering, Hanyang University, 04763, Seoul, Republic of Korea; Department of Nuclear Engineering, Hanyang University, 04763, Seoul, Republic of Korea; National Radiation Emergency Medical Center, Korea Institute of Radiological and Medical Sciences, 01812, Seoul, Republic of Korea; National Radiation Emergency Medical Center, Korea Institute of Radiological and Medical Sciences, 01812, Seoul, Republic of Korea; National Radiation Emergency Medical Center, Korea Institute of Radiological and Medical Sciences, 01812, Seoul, Republic of Korea

**Keywords:** retention function, internal dosimetry, thyroid dose, probabilistic estimation, uncertainty in bioassay

## Abstract

Bioassay functions, which are provided by the International Commission on Radiological Protection, are used to estimate the intake activity of radionuclides; however, they include considerable uncertainties in terms of the internal dosimetry for a particular individual. During a practical internal dose assessment, the uncertainty in the bioassay function is generally not introduced because of the difficulty in quantification. Therefore, to clarify the existence of uncertainty in the bioassay function and provide dosimetrists with an insight into this uncertainty, this study attempted to quantify the uncertainty in the thyroid retention function used for radioiodine exposure. The uncertainty was quantified using a probabilistic estimation of the thyroid retention function through the propagation of the distribution of biokinetic parameters by the Monte Carlo simulation technique. The uncertainties in the thyroid retention function, expressed in terms of the scattering factor, were in the ranges of 1.55–1.60 and 1.40–1.50 for within 24 h and after 24 h, respectively. In addition, the thyroid retention function within 24 h was compared with actual measurement data to confirm the uncertainty due to the use of first-order kinetics in the biokinetic model calculation. Significantly higher thyroid uptakes (by a factor of 1.9) were observed in the actual measurements. This study indicates that consideration of the uncertainty in the thyroid retention function can avoid a significant over- and under-estimation of the internal dose, particularly when a high dose is predicted.

## Introduction

Intake estimation after internal exposure to radioiodine, which is one of the main fission products in a nuclear power plant, is typically carried out using thyroid bioassay measurements and corresponding bioassay functions (i.e., thyroid retention functions). However, in addition to the measurement data, the thyroid retention function exhibits a significant uncertainty because of the lack of accurate knowledge on biokinetic models, inter-individual variability, and/or mathematical assumptions made for computational convenience [1]. The uncertainty in the thyroid retention function naturally introduces an uncertainty in the intake estimation, and consequently, in the internal dose estimation. In particular, in the absence of information regarding the intake time, a statistical fitting process with multiple measurements is required, and the uncertainty in the thyroid retention function can directly affect the fitting result. Therefore, considering the uncertainty in the bioassay function could help improve the accuracy of internal dose estimates.

In practice, the uncertainty in the bioassay function is not introduced in general data fitting processes because of the difficulty in quantification. For this reason, dosimetrists may misinterpret the bioassay data and fitting result. For example, if a clear information of the intake time (e.g., time-dependent air concentration) is given but the bioassay fitting result is statistically rejected, a dosimetrist may misadjust the intake time to improve the fitting result rather than trust the given intake time information. This problem can occur if the dosimetrist is unaware that the bioassay function involves significant uncertainty in the case of a particular individual. Another example is the use of thyroid measurement data obtained within a day after exposure. After internal exposure, thyroid measurement is acquired as soon as possible; hence, dosimetrists sometimes need to evaluate the internal dose using the data measured in the early phase. However, because the thyroid activity rapidly increases until a day after exposure [2], the thyroid activity in the early phase is unstable. In addition, in this time period, the assumptions of the first-order kinetics in the biokinetic model calculation may not be sufficient to simulate the rapidly changing thyroid activity and therefore can cause bias in the thyroid retention function; a similar problem was observed in the human alimentary tract model calculation [3]. Thus, early-phase thyroid data should be used with caution.

In this study, to clarify the existence of uncertainty in the thyroid retention function and give dosimetrists an insight into its uncertainty, the uncertainty in the thyroid retention function was quantified through the uncertainty propagation of the biokinetic parameters. Further, the uncertainty was numerically expressed in terms of the scattering factor to practically apply it to the maximum likelihood fitting method, which is the most widely used fitting method. Within a day after exposure to radioiodine, the uncertainty in the thyroid retention function was separately analyzed and the uncertainty due to the use of first-order kinetics was additionally quantified in terms of the bias, through comparison with the available measurement data.

## Materials and methods

**Fig. 1.**
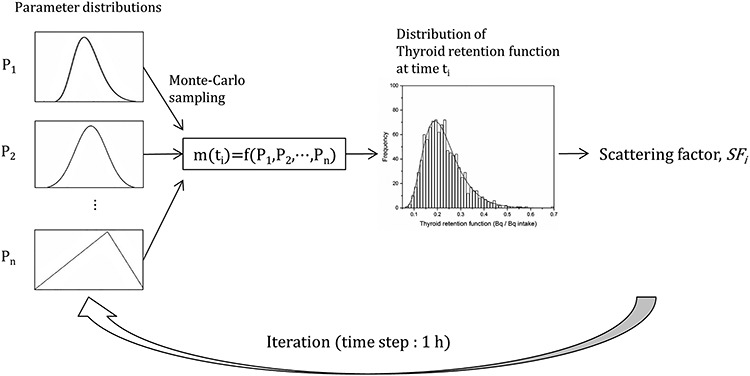
Schema for probabilistic estimation of the thyroid retention function. P_n_ and m(t_i_) represent the probability distribution of the n-th parameter and the thyroid retention function at time t_i_, respectively.

The uncertainty quantification of the thyroid retention function was implemented through its probabilistic estimation, whereby the radioiodine activity in the thyroid is predicted not as a single value but in the form of a probability distribution. The probability distribution was generated via multiple calculations of the thyroid retention function with a set of biokinetic parameters assigned using the Monte Carlo simulation technique from the corresponding parameter distributions. The probability distributions of the thyroid retention functions were produced with a time interval of 1 h, and the scattering factors as indicators quantifying the variances of the distributions were derived from each distribution at each time step. Figure 1 shows the schema for the probabilistic estimation of the thyroid retention function. The bias representing the uncertainty due to the use of first-order kinetics was quantified by comparing human-based measurement data with the corresponding calculated values.

### Maximum likelihood method for intake estimation

The principle of the maximum likelihood method for the bioassay has been explained in the IDEAS guidelines [4]. In the maximum likelihood method, the intake activity of radionuclide is determined as the value that maximizes the likelihood function *L(I)*. In general, the i-th likelihood function, *L_i_(I)*, is defined as follows:

(1) \begin{equation*} {L}_i(I)=P\left({M}_i/I\right) \end{equation*}

where *P(M_i_/I)* is the probability of observing the measurement data, *M_i_*, given the true value of the intake, *I*. However, to consider the uncertainty in the bioassay function, *L_i_(I)* should be expressed as in Eq. 2:

(2) \begin{equation*} {L}_i(I)=P\left({I}_e/I\right) \end{equation*}

where *I_e_* is the estimate of intake activity calculated using *M_i_/m_i_*, and *m_i_* is the bioassay function corresponding to *M_i_*. The probability distribution of M_i_, P(M_i_), represents the measurement uncertainty. The component of the measurement uncertainty can generally be divided into Type A (statistical) and Type B (non-statistical) errors. The Type A error is involved only in the counting statistics and is described by a Poisson distribution. The Type B error is involved in uncertainties other than the counting statistics and is generally described by a log-normal distribution; for example, the variability of the thyroid mass described by a log-normal distribution causes uncertainty in the counting efficiency, which is a Type B error. However, for simplicity, the overall uncertainty, P(M_i_), can be assumed to be a log-normal distribution [4]. This approximation is reasonable when the measurement counts are high and the Type A error is relatively low. For the probability distribution of m_i_, P(m_i_), representing the uncertainty in the bioassay function, the type of distribution can be determined by the uncertainty propagation of the biokinetic parameters, which are used to calculate the bioassay functions. Irrespective of the type of distribution used for the biokinetic parameters, the type of distribution of the overall uncertainty, P(m_i_), should be inductively determined from the final distribution in which the biokinetic parameter uncertainties are combined. In this study, P(m_i_) will be described by the log-normal distribution (see Figure 3). If both P(M_i_) and P(m_i_) can be described by log-normal distributions, as explained above, P(I_e_/I) may also exhibit a log-normal distribution, and the scattering factor (SF) can be defined as its geometric standard deviation. Therefore, L_i_(I) can be written as

(3) \begin{equation*} {L}_i(I)=\frac{1}{M_i\ln \left({SF}_i\right)\sqrt{2\pi }}\exp \left[\frac{{\left[\ln \left(\frac{M_i}{m_i}\right)-\ln (I)\right]}^2}{2{\left[\ln \left({SF}_i\right)\right]}^2}\right] \end{equation*}

where *SF_i_* is the scattering factor as a measure of the uncertainty in *I_e_*, which can be expressed as a combination of the measurement uncertainty, *SF_M_*, and the bioassay function uncertainty, *SF_m_*, as follows.

(4) \begin{equation*} {SF}_i=\mathit{\exp}\sqrt{{\left[\ln \left({SF}_{M_i}\right)\right]}^2+{\left[\ln \left({SF}_{m_i}\right)\right]}^2} \end{equation*}

Although laboratory-based *SF_M_* can be derived from the experimental conditions, typical values can be used; for example, 1.2 has been suggested as the typical *SF_M_* for high-energy gamma (>200 keV) [4]. Deriving appropriate values for *SF_m_* is the purpose of this study.

When *n* measurement data are independent, the combined likelihood function, *L(I)*, can be calculated by the product of the likelihood functions as in Eq. 5.

(5) \begin{equation*} L(I)=\prod_{i=1}^n{L}_i(I) \end{equation*}

Therefore, when *L(I)* is the maximum, the corresponding *I* is determined as the final intake estimate.

### Subjects for uncertainty quantification and default information

Table 1 presents the materials and compounds employed for the uncertainty quantification in this study and their corresponding default information. These were determined considering the available uncertainty information as follows: inhalation of elemental/inorganic iodine vapor; inhalation of organic iodine vapor; inhalation of aerosol particles of 1 and 5 μm AMAD; and ingestion of all iodine compounds. The corresponding default information for the bioassay function calculation has been provided in the ICRP publication [5]. The F-type clearance has been designated as the default absorption type for the inhalation of aerosol particles of iodine, and the elemental/inorganic iodine vapor and organic iodine vapor have been assigned to “SR-1” class with F-type and V-type, respectively. The fractional absorption, *f_1_*, for the ingestion of iodine was assumed to be 1. Although the thyroid retention functions were calculated for iodine-131, it is reasonable to apply the uncertainty quantification results to other iodine isotopes because only the biological parameters were considered as the uncertainty factors.

**Table 1:** Subjects for uncertainty quantification and default information

Intake route	Material type	Compounds	Default information
inhalation	Aerosol	All compounds	5 μm, F-type
	Aerosol	All compounds	1 μm, F-type
	Vapor	Elemental/inorganic iodine	SR-1, F-type, 100% deposition
	Vapor	Organic iodine	SR-1, V-type, 70% deposition
ingestion	Total-diet	All compounds	f_1_ = 1

### Calculation of thyroid retention function

The thyroid retention function can be calculated using the biokinetic models, which can mathematically describe the behavior of radioiodine after intake. The biokinetic models were first written as a set of first-order differential equations, as in Eq. 6, and then solved using matrix algebra introduced by Polig [6].

(6) \begin{equation*} \frac{\mathrm{d}{\mathrm{q}}_{\mathrm{i}}}{\mathrm{d}\mathrm{t}}=\sum_{\mathrm{j}=1,\mathrm{j}\ne \mathrm{i}}^{\mathrm{N}}{\mathrm{r}}_{\mathrm{i}\mathrm{j}}{\mathrm{q}}_{\mathrm{j}}-{\mathrm{q}}_{\mathrm{i}}\sum_{\mathrm{j}=1,\mathrm{j}\ne \mathrm{i}}^{\mathrm{N}}{\mathrm{r}}_{\mathrm{j}\mathrm{i}} \end{equation*}

where *q_i_* is the amount of iodine in the i-th compartment, and *r_ij_* is the transfer rate from j- to i-th compartment. In this study, the thyroid retention functions were calculated only for an acute intake that was more likely to happen in emergency situations. The calculation of the thyroid retention function was conducted using the computer code developed in an earlier work [7], which produced reliable thyroid retention functions and thyroid dose coefficients whose deviations from the ICRP reference values were within 3%. Therefore, the validation and verification of the computer code are not given herein.

### Biokinetic parameter distributions

Biokinetic models generally include a respiratory tract model for inhalation intake, an alimentary tract model for ingestion intake, and a systemic model for describing the iodine behavior after uptake to blood. Although the uncertainty in the biokinetic model originates from both model structure and parameter, only the uncertainty in the biokinetic parameter was considered in this study. The uncertainties in the biokinetic parameters for radioiodine are explained in detail in the following with data sources.

#### Respiratory tract model

The ICRP developed a human respiratory tract model (ICRP 66) [8]. Although partial modifications to this model have been recently made with new information in ICRP 130 [9], the ICRP 66 model was adopted in this work because the uncertainty information of the modified model was unavailable. In the ICRP respiratory model, the main mechanisms of the inhaled material are deposition in the lung and clearance from the lung. The distributions of the regional depositions for aerosol particles of 1 and 5 μm AMAD were referred from Harvey and Hamby (2001) [10] and Fritsch (2006) [11], respectively, and the data provided by Harvey and Hamby (2004) [12] were used for elemental/inorganic iodine vapor. The distribution of the total deposition of organic iodine vapor was referred from Morgan and Morgan (1967) [13] with the assumption that the data for methyl iodine could represent other organic forms. The distribution of deposition parameters are shown in Table 2. With regard to the clearance parameters in the lung model, the distributions of the fractional deposition parameters and rational clearance rate constants were obtained from Bolch et al. (2003) [14]. However, the uncertainties in the absorption parameters describing the uptake from the lung into blood were not considered in this work due to lack of information. The numerical uncertainty values of the clearance parameters taken from literature are not shown herein.

**Table 2:** Distributions of regional deposition fractions in the respiratory tract model taken from literature

Regions	Distribution^a^	Reference
*Aerosol, 1 μm*		
ET_1_	N (0.17, 0.035)	[10]
ET_2_	N (0.22, 0.045)	
BB	N (0.013, 0.0023)	
bb	LN (0.018, 1.21)	
AI	LN (0.10, 1.23)	
*Aerosol, 5 μm*		
ET_1_	LN (0.33, 1.2)	[11]
ET_2_	LN (0.41, 1.22)	
BB	LN (0.025, 1.61)	
bb	LN (0.014, 1.85)	
AI	LN (0.053, 1.6)	
*Vapor, elemental/inorganic iodine*		
ET_1_	LN (0.071, 1.31)	[12]
ET_2_	LN (0.28, 1.20)	
BB	LN (0.24, 1.44)	
bb	LN (0.38, 1.32)	
AI^b^	-	
*Vapor, organic iodide*		
ET_1_	LN (0.075, 1.28)	[13]
ET_2_	LN (0.29, 1.19)	
BB	LN (0.24, 1.44)	
bb	LN (0.36, 1.34)	
AI^b^	-	

^a^N: Normal (mean, standard deviation), LN: Log-normal (GM, GSD)

^b^The AI regional uptake is negligible for gas and vapor type

#### Alimentary tract model

The human alimentary tract model in ICRP 100 [15], which is the most recent model, was adopted, and the distribution to describe the uncertainties in the transfer rates was derived from the quantitative uncertainty factors (UFs) judged by Leggett et al. (2007) [3]. Leggett et al. (2007) addressed the overall uncertainties in the ICRP 100 model prediction and subjectively judged the reliabilities of the reference values of the transit times in terms of the UF. The UF represents a range of true values with respect to the geometric mean (GM) with a confidence interval of 95%; in other words, the UF corresponds to the number by which the GM is multiplied or divided to obtain the 95% confidence limit. Thus, the log-normal distribution is generally assumed when the UF values are provided with respect to the GM [14]. Therefore, in this study, the GM and geometric standard deviation (GSD) of the distribution of the transfer rates in the alimentary tract model were derived from the UF values with the assumed log-normal distribution, as shown in Table 3. For the fractional absorption, *f_1_*, of iodine, the triangular distribution with a mode of 1.0 and a range of 0.9–1.0 provided by D. M. Hamby (1999) [16] was used.

**Table 3:** Distributions of transfer rates in the alimentary tract model derived from uncertainty factors (UFs) suggested by Leggett (2007)

From	To	Distribution^a^
O-cavity	Oesophag-f	LN (6480, 1.52^b^)
O-cavity	Oesophag-s	LN (720, 1.52^b^)
Oesophag-f	St-cont	LN (12343, 1.52^b^)
Oesophag-s	St-cont	LN (2160, 1.52^b^)
St-cont	SI-cont	LN (20.57, 1.28^c^)
SI-cont	RC-cont	LN (6, 1.28^c^)
RC-cont	LC-cont	LN (2, 1.28^c^)
LC-cont	RS-cont	LN (2, 1.28^c^)
RS-cont	Faeces	LN (2, 1.28^c^)

^a^LN: Log-normal (GM, GSD)

^b^Derived from the proposed uncertainty factor of 2 for the 95% confidence interval

^c^Derived from the proposed uncertainty factor of 1.5 for the 95% confidence interval

#### Iodine systemic model

**Fig. 2.**
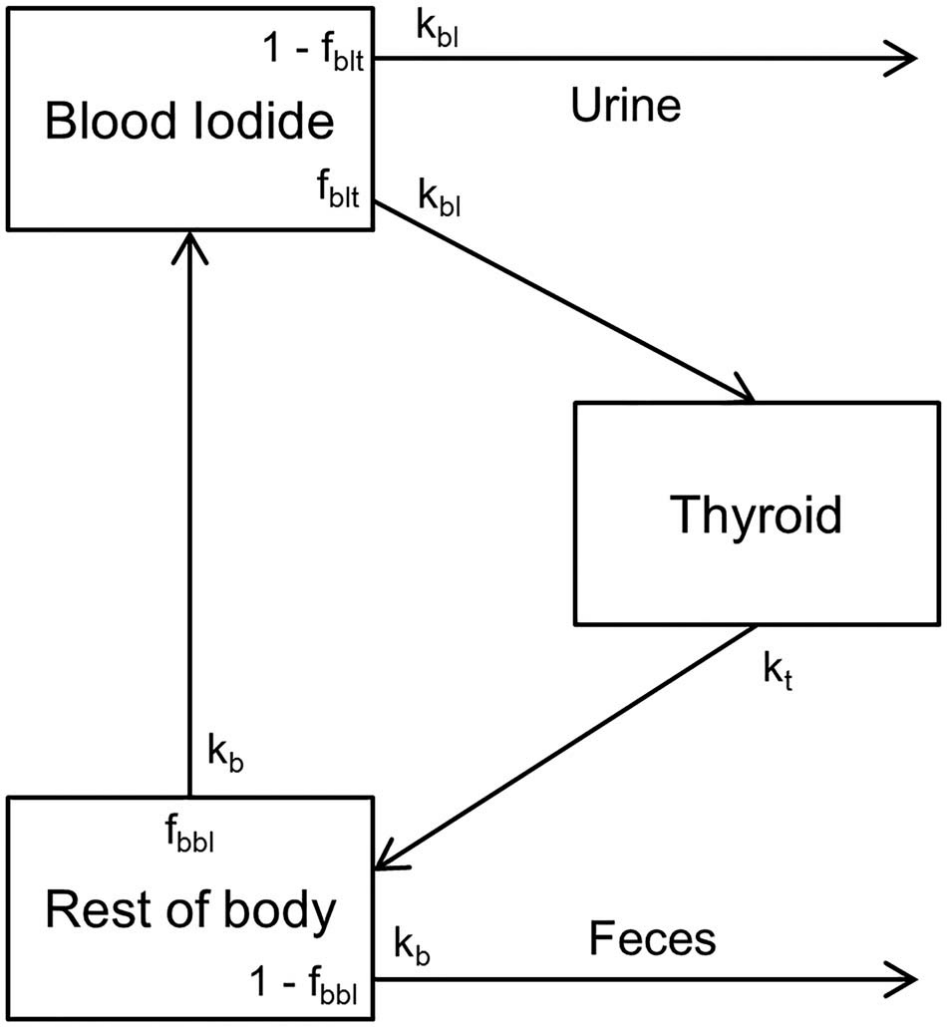
Three-compartment systemic model for iodine (ICRP 67)

The simple biokinetic model shown in Figure 2 (referred to as a three-compartment model hereafter) applied in ICRP publication 67 [17] as the primary biokinetic model has been used for many years to describe iodine behavior after uptake to blood. In this model, the iodine metabolism is described by the movements between the blood iodide, thyroid, and rest-of-body compartments. Recently, a new systemic model was developed by Leggett (2010) [18] and was used in the recent ICRP publication 137 [2]. Because this new model segmented the extra-thyroidal compartment into specific organs and assigned organic and inorganic iodine to these separate compartments, it could better describe the time-dependent iodine behavior. However, there are difficulties in applying the new model to this study. First, the uncertainty in the new model has not been investigated, and no information is currently available on the uncertainties of the model parameters. Second, and more importantly, the current internal dosimetry systems in most countries are mainly based on the ICRP publication 60 [19]; therefore, the thyroid retention functions calculated using the three-compartment model rather than the new ICRP model continue to be used. Even though the thyroid retention function calculated using the new ICRP model was published for occupational intake [2], it takes a considerable amount of time to apply the new thyroid retention function in real situations owing to regulatory and technical problems (e.g., application to dosimetry software), as well as other issues. Therefore, in this study, the three-compartment model was employed instead of the new model to provide practical understanding and useful data to dosimetrists.

The uncertainties in the transfer rates of the three-compartment model were investigated in various studies. In this study, the parameter distributions for the iodine systemic model were referenced from Hamby (1999) [16], as shown in Table 4. Hamby separately assigned proper distributions to the loss constant and fraction based on the collected data. The transfer rate can be calculated by multiplying the loss constant with the loss fraction. The sensitivity analysis conducted in that study indicated that the time-integrated thyroid activity was predominantly attributable to the thyroid uptake fraction (contribution of 95.7%); this means that the final distribution of the thyroid retention function would be dominantly determined by the thyroid uptake fraction described by a log-normal distribution. It should be noted that the distribution of the thyroid uptake fraction was derived from the distribution of the thyroid mass. Thus, the variability of the thyroid mass also has great importance in terms of the uncertainty in thyroid biokinetics.

**Table 4:** Distributions of biokinetic parameters in iodine systemic model from Hamby (1999)

Symbol	Parameter	Distribution^a^
f_blt_	Thyroid uptake fraction^b^	LN (0.267, 1.47)
f_bbl_	Body-blood fraction	T (0.823, 0.914, 1.000)
k_t_	Thyroid loss constant	T (67.8, 113, 158)
k_b_	Body loss constant	T (9.6, 12, 14.4)
k_bl_	Blood loss constant	T (0.0025, 0.25, 0.4975)

^a^LN: Log-normal (GM, GSD), T: Triangle (minimum value, mode, maximum value)

^b^The distribution of the thyroid uptake fraction was determined based on a mass-dependent uptake fraction of 0.015 per gram of thyroid.

### Monte Carlo technique

A set of biokinetic parameters for the multiple calculations of the thyroid retention function was assigned from each corresponding distribution using a Latin hypercube sampling (LHS) method, which is the most widely used method for Monte Carlo sampling. Because the LHS method draws samples from evenly divided probability intervals of a cumulative density function, the entire range of the distribution is used for sampling [20]. Thus, the LHS method can considerably improve the sampling efficiency. The sampling number (i.e., the multiple number of calculations) was determined to ensure a mean standard error of less than 2%. The computer codes for the Monte Carlo technique were developed in MATLAB R2015b [21].

### Uncertainty due to the use of first-order kinetics within a day after exposure

First-order kinetics were assumed for computational convenience. In the biokinetic model calculation with first-order kinetics, radioiodine can be removed only with constant half-life. However, the actual movement of radioiodine is more complicated, particularly in the early phase after intake. In this study, 24 h (1 d) was regarded as the time limit where the assumption of the first-order kinetics can introduce an uncertainty because after a day, most of the iodine is accumulated in the thyroid and eliminated gradually with a half-life of 80 d [5]. The uncertainty due to the use of first-order kinetics was quantified in terms of a bias. This bias is the magnitude of the discrepancy between the thyroid retention function and the actual measurement data. Because the correction of the thyroid retention function is not feasible in general dose assessment software, the correction can be made by dividing the thyroid measurement data, *M*, obtained within a day by the bias. Thus, the corrected thyroid measurement data, *M_corrected_*, can be calculated using Eq. 7.

(7) \begin{equation*} {M}_{corrected}=M/ Bias \end{equation*}

The bias was determined by comparing the measured and calculated radioiodine thyroid uptakes (%RAIU) within 24 h after iodine ingestion. Although the thyroid uptake data for intravenously injected iodine are required to clearly address the uncertainty related to the systemic model of iodine, the data obtained within and after 24 h in the same study were unavailable. Thus, the thyroid uptake after oral administration was used with the assumption that the mechanisms in the alimentary tract before iodine uptake to blood had little effect on the overall biokinetics of iodine. This assumption is reasonable because the absorption of iodide from the alimentary tract of humans is quite fast with a rate of approximately 5% min^−1^ and is completed within 2 h [22]. Therefore, the bias calculated in this study is deemed applicable to other intake routes such as inhalation, injection, as well as ingestion. The %RAIU measured within 24 h after oral administration of iodine for euthyroid subjects were collected from previous studies^(^ [23–29]^)^ and normalized to the value at 24 h. Thereafter, the normalized %RAIUs were compared with the corresponding values calculated using the first-order kinetics. The bias was derived as the ratio of the measured values to the calculated values. Note that because the reported data used in this study were measured by well-collimated systems or were properly corrected with the background counts, the uncertainty due to the extra-thyroidal radioiodine was negligible.

## Results

### Probabilistic estimation of thyroid retention function

**Fig. 3.**
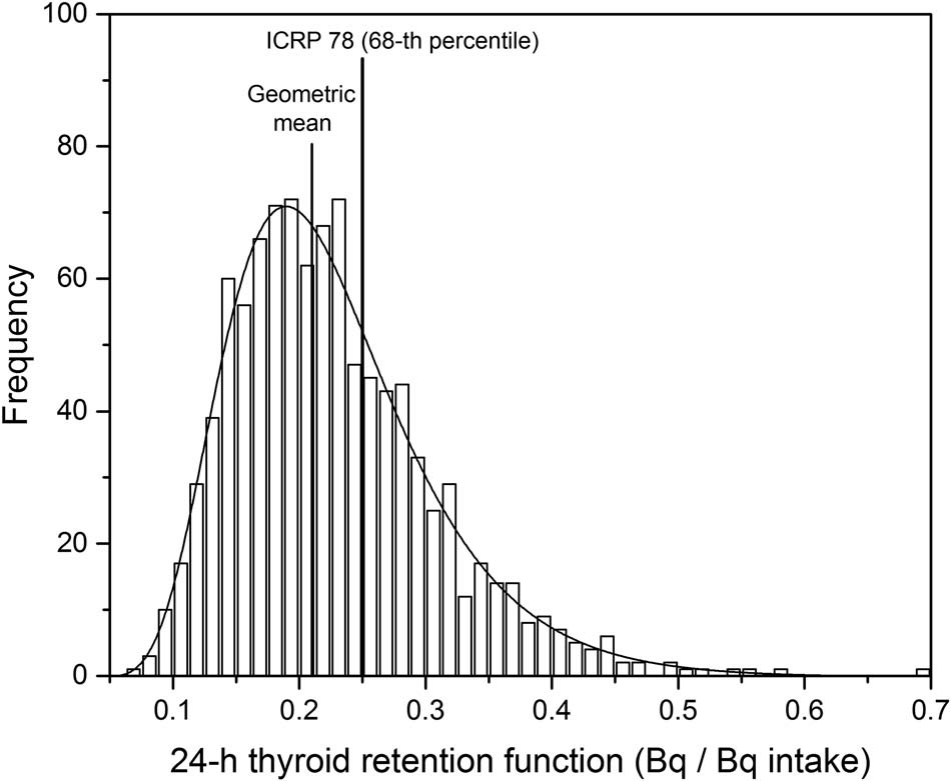
Distribution of thyroid retention function at 24 h

**Fig. 4.**
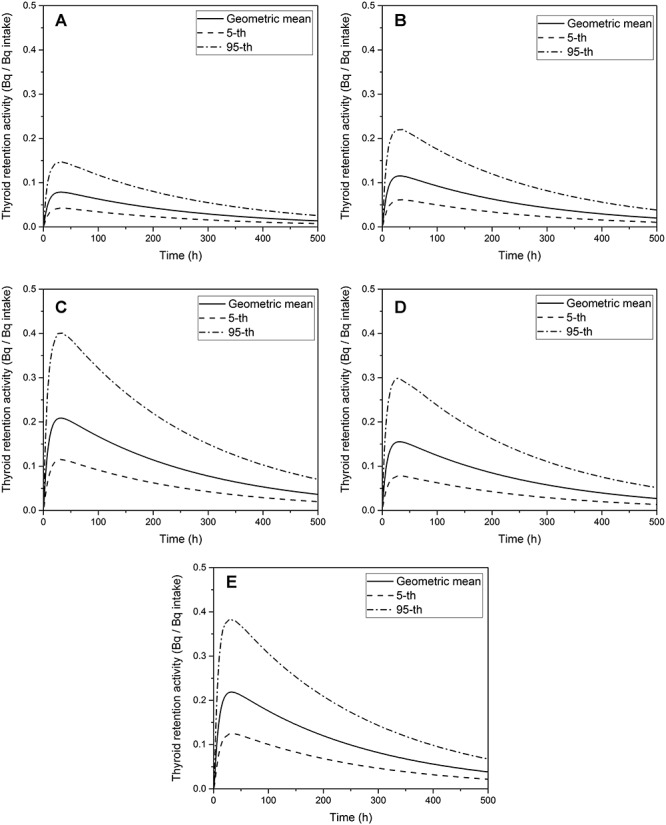
Probabilistic estimation of thyroid retention functions with median, 5-th, and 95-th percentiles: (a) inhalation of aerosol of 1 μm AMAD, (b) inhalation of aerosol of 5 μm AMAD, (c) inhalation of elemental/inorganic iodine, (d) inhalation of organic iodine, (e) ingestion of iodine

The probability distributions of the thyroid retention function were generated with increasing elapsed time after exposure to quantify the time-dependent uncertainty in the thyroid retention function. Multiple calculations with 1,000 sets of parameter samples at each time step reduced the mean standard error of the distribution to below 2%; however, further calculations did not significantly improve the distribution. When the thyroid retention function at a particular time was drawn as a frequency distribution, it was log-normally distributed. For example, as shown in Figure 3, the distribution of the thyroid retention function of iodine-131 at 24 h after ingestion of 1 Bq can be described by a log-normal distribution (R^2^ = 0.98) with GM of 0.21 and GSD of 1.41. The value of ICRP 78 [5] falls at the 68-th percentile of the distribution. The thyroid retention functions calculated for various intake routes and iodine compounds were drawn as the GM values with a confidence interval of 90% (i.e., the range of 5-th to 95-th percentile), as shown in Figure 4. In all the cases, the confidence intervals are the widest at approximately 24 h and tend to become slightly narrow with time.

### Scattering factors for describing the uncertainty in thyroid retention function

**Fig. 5.**
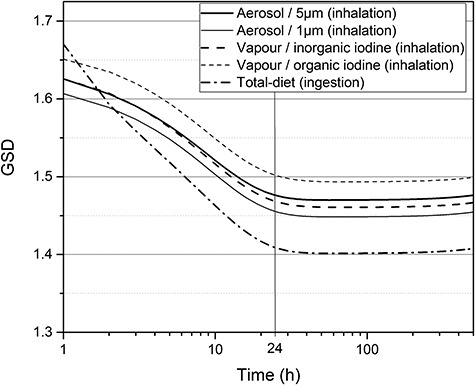
Time-dependent geometric standard deviations of the distributions of thyroid retention functions

Considering the shape of the distribution of the thyroid retention function shown in Figure 3, the scattering factor was defined as the GSD assuming that the uncertainty was distributed log-normally. Thus, the uncertainty quantification was implemented based on the time-dependent GSD of the distribution of the thyroid retention function shown in Figure 5. The GSD curves start at values higher than 1.6 and gradually decrease until 24 h. After 24 h, all the GSD curves reach their respective minimum values and do not change thereafter. In particular, for the ingestion of total dietary iodine, the decrease over time in the uncertainty is relatively significant. The GSD for the ingestion starts at the highest value, 1.7, and decreases to the lowest value, 1.4, within 24 h. Although the SF can be directly extracted from the figure, in this study, the SFs of the thyroid retention functions were suggested as representative values separately for the time before and after 24 h for practical convenience, as listed in Table 5. Considering that after an accidental exposure to radioiodine, the effort to measure the thyroid is made as soon as possible, the SFs before 24 h were suggested as the averaged GSDs within 10 h.

**Table 5:** Suggested scattering factors

Intake route	Physical or chemical type	Suggested scattering factor	
		before 24 h^a^	after 24 h
inhalation	Iodine aerosol of 5 μm AMAD	1.55	1.45
	Iodine aerosol of 1 μm AMAD	1.55	1.45
	Elemental/inorganic iodine vapor	1.55	1.45
	Organic iodine vapor	1.60	1.50
ingestion	Total-diet iodine	1.55	1.40

^a^Based on the averaged GSDs within 10 h after exposure

### Uncertainty due to the use of first-order kinetics

**Fig. 6.**
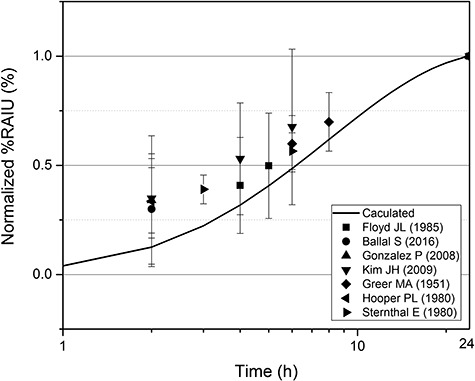
Comparison of the measured radioiodine uptake (normalized %RAIU) values and those calculated using first-order kinetics. The references given are to [23–29], respectively.

Figure 6 shows the normalized %RAIU values obtained from previous human-based experiments and those calculated using ICRP biokinetic models. The measured values are significantly higher than the calculated values. The ratio of the measured to the calculated values lies in the range of 1.13–1.94, and the average ratio across the data is 1.35. Therefore, data correction can be made by dividing the value measured within 24 h by 1.35, as described above.

### Examples for application of the uncertainty in thyroid retention functions

**Fig. 7.**
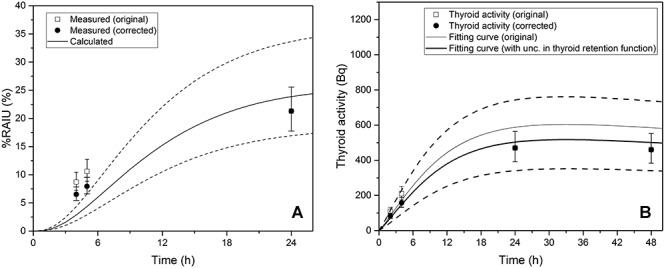
Examples for application of the uncertainty in thyroid retention functions: (a) comparison with actual measurement data (b) intake estimation based on artificial data

The scattering factors and bias suggested in this study were applied to examples in which thyroid measurements within a day after exposure were included. First, the uncertainty was applied to the data reported by Floyd et al*.* (1985) [23], in which the %RAIU for 142 euthyroid patients were measured at 4, 5, and 24 h after oral administration of iodine-123. Because the true intake was known (i.e., 100% of intake), the ability of the calculated RAIU curve derived from the thyroid retention function to well describe the measured values statistically (Figure 7(a)) was tested. When the calculated RAIU curve (solid line) fit to the original measured data (rectangular symbol), it was rejected (p < 0.05) because of the discrepancies in 4 and 5 h RAIU. However, when the scattering factors of the thyroid retention function are applied (dash lines) and the measured RAIU is corrected using the bias (circular symbol), the calculated RAIU curve is statistically not rejected (p > 0.05) even though the 4 and 5 h RAIU continue to show discrepancies. Although the calculated RAIU curve (solid line) seems to be adjacent to the value at 24 h, it is rejected (p < 0.05) because of the discrepancies in 4 and 5 h RAIU (rectangular symbol). However, when the scattering factors of the thyroid retention function are applied (dash lines) and the measured RAIU is corrected using the bias (circular symbol), the calculated RAIU curve is statistically not rejected (p > 0.05). In the second example (Table 6, Figure 7(b)), the artificial thyroid measurement data after inhalation of 5 μm iodine-131 aerosol are developed, and the intake estimation is carried out by data fitting using the maximum likelihood method. The intake was originally estimated to be 4,912 Bq with measurement uncertainty only; however, it was 4,208 Bq after considering the uncertainty in the thyroid retention function. Although both the fitting curves are not rejected (p > 0.05), the fitting curve considering the uncertainty in the thyroid retention function (bold line) is closer to the thyroid measurement value at 24 h.

**Table 6:** Example of uncertainty application (intake estimation based on artificial data)

	Original method		Application of uncertainty	
**Measurement data**				
Time (h)	Thyroid activity (Bq)	SF_M_	Corrected thyroid activity (Bq)	Combined SF ^a^
2	110	1.2	83	1.6
4	210	1.2	158	1.6
24	470	1.2	470	1.5
48	460	1.2	460	1.5
**Result**				
Intake estimate (Bq)	4,912		4,208	
p-value	0.087		0.982	

^a^Combined value of SF_M_ and SF_m_

## Discussions

The determination of the type of uncertainty distribution is critical for interpreting and quantifying the uncertainty. The type of distribution of the thyroid retention function was inductively determined after propagation of the biokinetic parameter uncertainties. The log-normal distribution selected for explaining the distribution of the thyroid retention function is statistically valid and corresponds with the results of the other studies; for example, the time-integrated activity of ingested iodine in the thyroid, which was calculated by integrating the thyroid retention function, was also described by the log-normal distribution [16]. Based on the sensitivity analysis conducted by Hamby [16], it could be presumed that the log-normal distribution of the thyroid uptake fraction played a main role in shaping the distribution of the thyroid retention function. Although Figure 3 shows only the case of 24-h thyroid retention function, all the distributions at each time step were log-normally distributed. Therefore, it is reasonable to regard the distribution of the thyroid retention function as a log-normal distribution and to quantify the uncertainties by their GSD.

Although measurement uncertainty has been considered in general bioassay data fitting methods, this study showed that the thyroid retention function also exhibits a significant uncertainty. Considering the typical SF of 1.2 for the measurement uncertainty of high-energy gamma, as suggested by the IDEAS guidelines, the major uncertainty can be attributed to the uncertainty in the thyroid retention function, whose SF values are higher than 1.4. In particular, the magnitude of the uncertainty was relatively large in the thyroid retention function within a day after exposure. Because the thyroid activity in the early phase after exposure is influenced by various factors, such as the amount of iodine in blood, blood-to-thyroid transfer rate, and thyroid-to-rest of the body transfer rate, it is difficult to predict the thyroid activity before the early thyroid uptake is complete. In addition, this study showed that the thyroid retention function calculated using the first-order kinetics could underestimate the actual thyroid uptake within a day after exposure. Although sufficient measured data were unavailable, obvious discrepancies between the thyroid measurement data and the calculated values were observed. There could be other reasons for the discrepancies, such as the extra-thyroidal radioiodine contribution and other factors that cannot be identified clearly. However, we judged that the influence of other factors was minor and negligible compared to the uncertainty from the use of first-order kinetics.

The effects of the uncertainty in the thyroid retention function were demonstrated in the previous examples. In the first example in which the true intake was known, applying the scattering factor and bias suggested herein made a difference in the statistical explanation ability of the calculated %RAIU curve. The correction using the scattering factor and bias produced p-value higher than 0.05. However, it should be noted that despite the statistical acceptance of the fitting, the discrepancy in the early data at 4 and 5 h indicated that the thyroid retention function in the early phase after intake would still involve a significant uncertainty. In the second example in which artificial measurement data were used, it was shown that the intake estimate could significantly vary depending on whether the uncertainty in the thyroid retention function was considered or not. In the example, the intake estimates changed by approximately 17%. In particular, the overestimation of the intake could be avoided by scaling down the data at 2 and 4 h, and the fitting curve became closer to the data after 24 h. From the results, it can be concluded that applying the uncertainty in the thyroid retention function reduces the importance of the unstable thyroid measurement data obtained within a day, but strengthens the relative reliability of the data obtained after a day. Depending on the situation, considering the uncertainty can also reduce the possibility of underestimation. If the exact time of intake is unknown, applying the bias can estimate an intake time later than that without the bias and thus result in a higher intake estimate.

The best way to avoid the uncertainty from the unstable measurement data in the early phase is by measuring the thyroid after 1 d of exposure. If only data measured after 1 d are used, the uncertainty is relatively low, and the result of intake estimation using the bioassay data fitting may not change because equal weightings are induced to all the measurement data regardless of the time. In this case, introducing the uncertainty in the thyroid retention function to the data fitting can only affect the good of fit, and thus the determination of acceptance or rejection of fit. Moreover, the thyroid measurement after 1 d is preferred because it can reduce the background effect of extra-thyroidal iodine on the thyroid measurement. Until radioiodine is absorbed into the thyroid or excreted via urine, significant amounts of radioiodine distributed to organs or tissues other than the thyroid can influence the thyroid measurement counting. Nevertheless, in actual situations, dosimetrists often need to perform intake estimation using only the data obtained within a day or data obtained both before and after a day. The first dose assessment should not be delayed after internal exposure. Therefore, the time-dependent uncertainty is vital for practical dose assessments and should be considered particularly important when early measurement data are used.

It is also important to clarify the exposure categories and situations where the uncertainty investigated in this study could be applied in practice. ICRP publication 103 [30] has classified the exposure situations as existing, planned, and emergency exposure situation, and the exposure categories as occupational, public, and medical exposure. Regarding the exposure situation, it is reasonable to apply the uncertainties only to the “emergency exposure situation,” where high dose is predicted and the accuracy of dose estimation is important for decision related to subsequent action. Because a higher dose involves greater uncertainty, efforts to reduce the uncertainty in dose assessment should be proportionate to the dose. For the other situations (i.e., existing and planned exposure situation), where relatively low dose is predicted, it may be preferable not to consider the uncertainty for the harmonization of dose estimation and regulatory consistency. However, it is advisable to not limit the exposure categories (i.e., occupational, public, and medical exposure) for application of uncertainty. If very high exposure is predicted and the bioassay data are available, it is necessary to consider the uncertainties in the thyroid function regardless of the exposure categories. For example, the exposure in routine activities and that in emergency activities after a nuclear accident may be categorized as “planned exposure situation” and “emergency exposure situation,” respectively; thus, uncertainty is considered in the latter case but not in the former case.

This study attempted to quantify the uncertainty in the thyroid retention function for practical applications. More importantly, the objective was to provide insights to dosimetrists into the uncertainty in the thyroid retention function rather than giving the exact numerical values. Although the uncertainty is not considered numerically, the dosimetrist should understand the extent of uncertainty in the thyroid retention function and thus be able to practically judge which data are more important for intake estimation. Because the internal dose calculation depends on the judgment of the dosimetrist, a firm understanding of the uncertainty is essential to derive more accurate dose estimates.

However, it should be noted that this study does not guarantee that applying the uncertainties suggested herein would always yield a more accurate dose estimate. Because the dose assessment needs to be based on a comprehensive understanding of the given data and conditions, dosimetrists should focus on the use of uncertainties depending on the situation.

## Conclusion

This study revealed the uncertainty in the thyroid retention function based on the Monte Carlo method and performed a comparison with actual measurement data. Moreover, the study provides new insights to dosimetrists into the uncertainty in the thyroid retention function and methods of applying it to practical dose assessments. The thyroid retention functions were assumed to be independent of one another; the accuracy of the uncertainty quantification can be improved by better understanding the correlation between the thyroid retention functions and through the development of a more extensive database. In addition, the use of recently developed biokinetic models for iodine can improve the accuracy of the thyroid retention function. This study lays a foundation for the uncertainty quantification of bioassay functions. Future studies should be directed toward quantifying the uncertainty in various bioassay functions for chronic intakes and for other radionuclides.

## Conflict of Interest

The authors have no conflicts of interest to declare.

## Funding

This research was supported by a grant of the Korea Institute of Radiological and Medical Sciences (KIRAMS), funded by Ministry of Science and ICT (MSIT), Republic of Korea. (No.50445-2020).
